# The Effect of an Anti-Bullying Intervention on Male Students' Bullying-victimization Behaviors and Social Competence: A Randomized Controlled Trial in Deprived Urban Areas

**Published:** 2019-10-21

**Authors:** Nooshin Salimi, Akram Karimi-Shahanjarini, Forouzan Rezapur-Shahkolai, Behrooz Hamzeh, Ghodratollah Roshanaei, Mohammad Babamiri

**Affiliations:** ^1^Department of Public Health, School of Public Health, Hamadan University of Medical Sciences, Hamadan, Iran; ^2^Social Determinants of Health Research Center, Hamadan University of Medical Sciences, Hamadan, Iran; ^3^Research Center for Health Sciences, Hamadan University of Medical Sciences, Hamadan, Iran; ^4^Research Center for Environmental Determinants of Health, Kermanshah University of Medical Sciences, Kermanshah, Iran; ^5^Department of Biostatistics, Modeling of Noncommunicable Disease Research Canter, Hamadan University of Medical Sciences, Hamadan, Iran; ^6^Department of Ergonomics, School of Public Health, Hamadan University of Medical Sciences, Hamadan, Iran

**Keywords:** Violence, Bullying; Schools, Health education, Iran

## Abstract

**Background:** Violence among adolescents is a global public health concern. There is limited evidence on the effectiveness of anti-bullying interventions in Iran. Weaimed to examine the effectiveness of social cognitive theory (SCT)-based intervention on reducing bullying and victimization in elementary school students.

**Study design:** A randomized controlled trial.

**Methods:** Eight elementary schools (consisted of 280 students in 5^th^ and 6^th^ grade) from deprived and semi-deprived areas of Kermanshah (west of Iran) were randomly assigned to intervention and control group from 2018 to 2019. Anti-bullying training content appropriate for SCT and sociocultural characteristics were provided to the intervention group including students, parents, teachers and school staff. The measures included SCT constructs, bullying, victimization, and social competence of students.

**Results:** At baseline participants of two groups were homogenous in terms of demographic factors except for the type of living with the parent (*P*=0.040) and outcome variables including SCT constructs and bullying behaviors. The interventions significantly improved SCT constructs, reduced bullying and victimization and increased social competence in the intervention group compared to the control group (*P*<0.001). The difference between outcome expectations in both groups was not significant (*P*=0.137).

**Conclusion:** Interventions based on sociocultural characteristics and focuses on SCT theory reduce bullying and victimization behavior. Given the effectiveness and feasibility of these interventions, this theory can be effective to break the bullying cycle and improve social competence.

## Introduction

The transition from childhood to adulthood is accompanied by complex psychological, physiological and social changes. These changes can expose adolescents to violence ^1^. Violence among adolescents is a global public health concern ^2^. Bullying is a type of violence in the school that typically occurs by a student or a group of students intentionally and repeatedly and by using force to impose physical or psychological damage to other students ^3^. The prevalence of bullying in the schools of different countries varies from less than 10% to more than 65% among children and adolescents^2^. In Iran, 39% of students had moderate and high aggressive behaviors and 75% of them were victimized in moderate and high levels ^4^ .

There are several forms of bullying, including physical bullying (like kicking, beating), verbal bullying (threatening and insulting) and social bullying (rejection and gossiping)^5^. Bullying and victimization can have permanent effects on the children and adolescents, which persists until adulthood^6^ like leaving the school, physical damages, social and psychological problems such as depression, anxiety, suicide thoughts and behavior, grief and sorrow, humiliation, lower self-rated health and reduction of self-esteem ^7-9^. Children and adults who lack enough social competence are more probably suffer social problems including bullying. On the other hand, the amount of bullying impacts the social competence of children, such that bullying is associated with lower social competence ^10^.

In recent decades, various studies have been conducted on bullying resulted in the implementation of intervention programs around the world. A wide range of effectiveness of these interventions makes it difficult to identify the most effective component in reducing the bullying among students ^11, 12^. For example, in a meta-analysis of 13 studies assessed the effectiveness of school-based antibullying interventions. This kind of interventions has a small to moderate effect on victimization. They also reported a significant heterogeneity across studies of victimization^12^.

Although the likelihood of reducing bullying in interventions that encompass different socio-ecological aspects of students (including interpersonal relationships, classroom and, school environment) is more than interventions that consisted of only one or two aspects ^12^.

There is little evidence about anti-bullying interventions in Iran and most researches were focused on the descriptive and relational studies or only emphasized one aspect of effective factors on bullying ^13, 14^. National initiatives launched to screen the violence among students in recent years are integrated into the social harms reduction programs, indicating there is not yet a comprehensive plan by related bodies such as the education department to prevent and fight against bullying among students.

Esteki Azad et al study is among rare anti-bullying interventions conducted in Iran. They measured outcome variables using Peer Relationship Questionnaires (PRQ) and reported that the intervention could reduce bullying and victimization among elementary school boys ^15^. In another Iranian anti-bullying study, an integrated anti-bullying intervention measured the students’ victimization behaviors. It offered a multi-targeted intervention to students, teachers, and school staff. The anti-bullying program was successful in reducing the verbal, physical, and emotional aspects of victimization in experimental schools (*P*<0.05)^16^. In the context of behavior changes, applying the theoretical framework are along with more successful ^17^. Social cognitive theory (SCT) is a well-known theory for understanding the social nature of bullying behaviors and the complexity of relationships between different factors. This theory shows the interaction between environment (for example, observing other people behavior), internal stimulants (for example, recognition and feelings) and behavior ^18^.

Given the high prevalence of bullying in Iran's schools and the lack of a proper anti-bullying program, we aimed to assess the effectiveness of a multi-component, culturally sensitive, and multi-targeted intervention to reduce the bullying and victimization behaviors as well as social competence of students.

## Methods

This randomized controlled trial study was conducted in elementary schools of deprived and semi-deprived areas of Kermanshah City, west of Iran from 2018 to 2019.

The study was approved by the Ethical Committee of Hamadan University of Medical Sciences (IR.UMSHA.REC. 2018.638). The informed consent form was obtained from students' parents.

The Iranian educational system at the elementary level comprises three years of lower elementary (1st to third grade) and three years of upper elementary (4^th^ to 6^th^grade). Kermanshah is a capital city of Kermanshah Province located in the west of Iran. It has a population of about 1.1 million residents ^19^. Totally, there are 263 elementary schools with about 77000 students in Kermanshah City. A power calculation was conducted to determine the sample size needed to detect a difference of 8% in the mean score of students' bullying behaviors ^20^. We set the alpha level at 5% and beta level at 10%. By adjusting the sample using a design effect of 1.5% and a 10% projected attrition rate over the follow-up, a total of 280 students were necessary (140 students in each of the intervention and comparison groups(.

Because of the nature of the intervention, we selected the elementary schools as the cluster unit. To ensure the homogeneity of the schools assigned to the groups, two medium schools (300 to 600 students) and two small schools (200 to 300 students) were considered for each group. Eligibility criteria for the schools were as follows: being elementary boy's school and locating in deprived and semi-deprived areas of Kermanshah. Given the average number of students in each class of schools located in the region of study and due to executive considerations, we recruited the sample from eight schools. Therefore, 8 boy's elementary schools were randomly selected to participate in the study from a sampling frame of all eligible schools (n 41). After stratification by school size, each school was randomly assigned to the intervention group (n=4) or the control group (n=4) by drawing lots (concealed from participants; 1:1 allocation ratio). We conducted our study among fifth and sixth-grade boy students because a recent study conducted in schools located in the low- income areas of Kermanshah showed that these students were much more likely to engage in bullying behaviors^4^. Therefore, one class of 5^th^ or 6^th^ grade from each school was randomly selected to participate. All students in the selected classes were invited to take part in the study. Outcome measures consisted of SCT constructs, bullying, victimization behaviors, and social competence. *To avoid the impact of the students' readability* differences, the interviewer read all questionnaires aloud and then, students respond. The data were collected during school hours and it took about 40 min.

Students' questionnaire included four sections. The first section focused on the basic and demographic information including age, the number of children in the family, type of living with parents, mothers' occupation and the average daily time spent playing mobile and computer games. The second section consisted of SCT constructs items developed using previous studies and research team experiences ^4^. This section included knowledge about bullying and its consequences with 11 items (for example: is the gossiping about other people a bullying behavior?), self-efficacy to control bullying behavior with seven questions (for example, I can forgive someone who has annoyed me), social support to control bullying with seven items (for example: our teacher talk about the bad effects of bullying for us), perceived social norms in bullying with 12 items (for example: my parents believe that whenever it is necessary, I am allowed to sulk), observational learning with 10 items (for example, when my friends shouted during fight, I'll do it as well.), outcome expectations and outcome expectancies with five items (for example, the bullying behavior makes my friends fear me. / It's important for me that others fear me) and perceived situational with seven items (for example: I can report bullying in the school comfortably). The content validity of the questionnaire was evaluated by an expert panel consisted of 15 specialists in health education and promotion and one specialist in psychology. The internal consistency reliability was measured using Cronbach alpha. CVR, CVI, and Cronbach alpha coefficient of constructs obtained acceptable values: social support: α= 0.72, CVR= 0.93, CVI 0.98; self-efficacy: α= 0.77, CVR= 0.95, CVI= 1.00; observational learning: α= 0.87, CVR= 0.89, CVI 0.96; perceived social norms: α=0.81, CVR= 0.88, CVI 0.96; outcome expectancies: α=0.72, CVR=0.88, CVI 1.00; outcome expectations: α=0.70, CVR 0.88, CVI 1 ; perceived situational: α=0.73, CVR= 0.86, CVI 0.95.

Bullying was measured using the Adolescent Peer Relations Instrument (APRI) ^21, 22^. That is a multidimensional measure and has been used in previous studies conducted in Iran ^23^. The Iranian version of APRI could explain 44.83% of the total variance and the reliability of the bully and victim factors were 0.92 and 0.92, respectively ^23^. APRI addresses how often the children and adolescents had been a victim of bullying or have bullied others on a six-point scale (1=never to 6=every day). This 36- item tool measures the different aspects of bullying (including 18 items: verbal, social, and physical bullying) and, victim (including 18 items: verbal, social, and physical) behaviors of children and adolescents. To create an overall scale of bully and victim behaviors, we summed responses to items of each dimention.

The fourth section of the questionnaire was devoted to the measure of students' social competence by a 44- item version of Rosman and Kohn social competence ^24^. The teachers asked to complete the questionnaire considering the students' interaction with others. This scale involved two subscales: Interest-participation versus apathy-withdrawal and cooperation-compliance versus anger-defiance. Each item has an answer with the five-point scale from always to never that score five is considered for always and score one for never.

The intervention targeted at multi groups involving students, parents and teachers/school staff Developed to improve the SCT variables, students' skills, and their social competence and effective relationships to reduce the involvement in bullying behavior and victimization applying the concepts of the whole school approach^2^ and behavior change strategies ^17^ in the shade of the local cultural values (Table 1).

**Table 1:** educational Programs to control and reduce bullying in elementary students

**Target construct**	**Practical Applications**
Knowledge about bullying	● Booklet ● Speech ● Discussion ● Question & answer
Self-efficacy to control bullying	● Modeling (role-playing to control bullying) ● Verbal persuasion (teachers, school staff & parents were asked to encourage the students who control their bullying) ● Conducting an action in small steps (step by step training for anger control by students, parents, teachers and school staff)
Social support	● Networking (forming a telegram group for parents and another group for teachers and school staff) ● Enhancing the network (providing suitable messages in the telegram group of parents, teachers, school staff and get feedback) ● Enhancing the friendly relationship in students through friend-finding skills training ● Providing emotional support (paying attention to students' problems) ● Providing information support (speech & group discussion about the definition of bullying, its types, its side-effects and bullying control methods) ● Providing instrumental support (providing booklet, poster, referring bully students to consultation center freely)
Observational leaning & perceived social norms	● Group discussion and role-playing about right and wrong beliefs related to bullying and ethical conclusion by students ● Training parents, teachers and school staff about their importance as role-model for students and controlling their violent behaviors.
Outcome expectation & outcome expectancies	● Group discussion about the values and negative consequences of bullying and positive results of anger control for students, parents, teachers & school staff ● Writing a memoir about bullying & its consequences & ethical conclusion by students ● Performing 5 designed scenarios by students once with bullying & once with problem-solving and anger control
Perceived situational	● Resolving misconception through discussion (correct communication with teachers and school staff, reporting bullying by students) ● Providing suitable solutions for teachers & school staff (more monitoring on students in classroom and playground) ● Enhancing students relationships through teamwork and friendly games

Following assessing the existing educational material, we used some of them in the intervention and designed some other materials, including a booklet, two posters, texts and messages for speech, and five scenarios of role-playing. Prior to RCT, all of *intervention materials* were tested and necessary suggested corrections were applied.

Interventions were presented in four training sessions for six weeks for students. Four 20 min sessions were held for teachers and school staff, and one 90-min training sessions held for parents.

In order to control Hawthorne effect, along with providing the anti-bullying intervention to the intervention group, an intervention was done with a similar intensity and schedule with an unrelated topic (preventing the lice) for the control group. Kolmogorov-Smirnov test confirmed data normality. In order to compare groups before and after intervention, data were analyzed by independent t-test, paired t-test and chi-square test using SPSS 16 software (Chicago, IL, USA) and 5% significance level.

## Results

We had not attrition in both groups. According to reading one by one, of the questions by the questioner and assurance of students' responses, the number of missing data were low (less than 5%) that replaced with mean of other responses.

The study of demographic and contextual variables in both groups showed that the students of both groups were similar in all variables except their type of living with the parent at baseline (*P*=0.040) (Table 2).

**Table 2:** demographic variables in the intervention and control groups

**Variables**	**Intervention group**	**Control group**	***P*** **-value**
Age (yr)			0.103
10	2	5	
11	56	53	
12	75	81	
13	7	1	
Number of children in the family			0.088
1	32	19	
2	59	78	
3	25	30	
≥4	24	13	
Living with parent			0.040
With parents	119	131	
Mother or father	12	4	
None of them	0	2	
Time spent playing mobile/computer games (hr)			0.145
<1	29	35	
1-2	40	31	
2-3	26	24	
3-4	18	13	
>4	20	28	
Mother's employment			0.856
Employed	11	12	
Unemployed	123	124	

Table 3 shows the mean score of constructs, bullying, victimization and social competence at baseline and five months after intervention. There was no significant difference between bullying and victimization score, social competence, and SCT constructs between intervention and control groups at baseline. The results of independent t-test showed that significant improvement after the intervention in constructs like knowledge, self-efficacy, social support, perceived social norms, observational learning, outcome expectancies and perceived situational in the intervention group compared to control group (*P*<0.001). However, the mean scores of outcome expectations had not significant difference in both groups (*P*=0.137). The mean score of bullying and victimization in the intervention group had significant reduction and social competence had significant increase compared to the control group after the intervention (*P*<0.001).

**Table 3:** Comparison of the mean and standard deviation of social cognitive theory’s structures and behaviors, before and after the intervention among students in intervention and control groups

**Variables**	**Baseline**					**5-month follow-up**				
	**Control** **group**		**Intervention** **group**			**Control** **group**		**Intervention** **group**		***P*** **value**
	**Mean**	**SD**	**Mean**	**SD**	***P*** **value**	**Mean**	**SD**	**Mean**	**SD**	
Knowledge	5.51	2.32	5.49	2.22	0.916	5.71	2.10	7.55	2.32	0.001
self-efficacy	21.39	7.07	21.06	7.83	0.713	21.49	6.39	27.42	7.19	0.001
Social support	23.21	5.86	22.36	5.42	0.165	22.78	4.81	26.11	5.14	0.001
Perceived social norms	29.07	7.68	29.18	7.83	0.908	31.14	7.29	23.78	9.51	0.001
Observational learning	25.64	7.28	24.53	7.18	0.199	26.67	7.70	20.62	9.21	0.001
Outcome expectancies	18.60	4.36	17.80	4.54	0.134	18.36	3.98	15.51	5.01	0.001
Outcome expectations	14.48	4.81	14.14	4.54	0.540	13.93	4.22	12.97	6.31	0.137
Perceived situational	16.49	3.58	16.59	4.22	0.819	15.84	3.83	20.40	4.67	0.001
Physical bullying	16.93	7.30	15.34	6.55	0.056	17.66	7.41	12.21	6.60	0.001
Verbal bullying	13.93	6.23	14.38	6.28	0.548	14.82	6.38	11.56	6.39	0.001
Social bullying	13.32	5.39	13.38	6.74	0.938	14.01	5.90	10.61	6.04	0.001
Physical victimization	12.77	5.61	12.11	5.91	0.336	13.44	5.25	10.57	5.64	0.001
Verbal victimization	13.02	6.33	14.01	6.48	01.99	15.19	6.80	11.66	6.71	0.001
Social victimization	11.89	5.41	11.76	5.71	0.855	12.42	5.32	10.11	5.11	0.001
Total bullying	44.19	17.43	43.09	17.86	0.605	46.84	18.06	34.39	17.79	0.001
Total victimization	37.68	16.37	37.87	16.30	0.921	41.05	15.96	32.34	16.17	0.001
Social competence	-6.11	20.13	-2.94	22.53	0.215	-7.44	17.39	0.68	27.22	0.001

## Discussion

We aimed to assess the effectiveness of a theory-based intervention on reducing bullying and victimization in male students of elementary schools in Kermanshah-Iran. Educational intervention effectively reduced bullying and victimization behaviors in a deprived and semi-deprived area. The intervention was based on sociocultural characteristics and a brief program that increase the likelihood of its feasibility and acceptability in the educational system in Iran. Results of this study are consistent with results obtained from some previous studies ^20, 25^. For example, an intervention including influencing the peer group, creating supportive environments, and strong networks of personal attachments could reduce all types of bullying in students ^25^. Other similar studies have reported promising results in reducing bullying behavior among students applying a multi-component intervention to address influencing factors and by involving key stakeholders such as students, parents, and teachers ^26, 27^.

Along with bullying and victimization behavior, we assessed the effect of the intervention on students’ social competence. Our results showed significant increase in the intervention group compared to control group. Intervention based on reducing bullying can lead to improvement and increase in the social competence of students. Aggression Replacement Training (ART) could increase the social skills of students and decrease their behavioral problems ^28^.

There is a relationship between knowledge about bullying and bullying behaviors in students suggesting that a student with lower knowledge is more intended to involve in bullying behavior ^29^. In our study, the knowledge of students of intervention group improved significantly. Concurrent use of booklet and poster, lecture, and question-answer sessions were effective in increasing the knowledge of students. Saibon et al increased the knowledge of students about bullying by using a creative and artistic training method including poetry, music, question-answer sessions and booklet ^29^. Moreover, while the mean score of outcome expectancies reduced significantly in the intervention group, reduction in outcome expectation on bullying was not significant. We used role-playing and writing memoire to make change in these constructs. Amse et al modified the outcome expectations on bullying in through performing a theater and group discussion^30^. The enhancement of this construct needs more time. The difference in follow-up time can be important in the difference of the results because the methods used in Amse study were similar to our methods except the follow-up in their study began immediately but it was done after five months in our study. Self-efficacy that refers to individual belief about his/her ability to do a behavior. Self- efficacy is one of the most important preconditions of changing behavior ^31^. Our intervention was successful in improving the students’ self- efficacy. The intervention addressed this factor by modeling, verbal persuasion and acting in small steps were done to promote the self-efficacy of students. Training intervention increased the self-efficacy of students in defending victims^30^. SCT emphasized observational learning to enhance the self-efficacy in complex behaviors ^17^. Observational learning plays an important role in bullying by children and adolescents. Those who are exposed to bullying and other aggressive behavior are more likely to participate in bullying behavior ^18^. After the intervention, the mean score of observational learning reduced significantly in the intervention group compared to the control group. To improvement this construct, the students were asked to play their scenarios once with bullying and once with problem-solving and anger control, and finally, present their ethical conclusion. By providing suitable training, teachers and parents were asked to prevent violence as the role-model of students. In this regard, Laspata et al reduced aggressive behaviors in elementary students through video self-modeling^32^. Based on SCT, observational learning does not lead to behavior change without the support of the environment ^17^. According to national school climate council, school climate is a perception of the people of goals, values, norms, interpersonal relationships, teaching and learning methods and organization structure that keep people safe socially, emotionally and physically ^33^. In the current study, after the intervention, the perceived situational of students from school climate showed a significant increase in the intervention group compared to the control group. In this study, we tried to improve this construct by improving the attitude of students about coping with bullying and expressing positive consequences of coping with bullying, presenting suitable solutions for teachers and school staff such as more monitoring on students in class and playground, enhancing the relationships of students through teamwork and friendly games. Ferrer-Cascales used peer tutoring in their study to improve school climate, including satisfaction with school, participation and positive relationship between the school and family, and sense of belonging to school. The students with high interpersonal skills were responsible to help their peers after receiving training. Ferrer-Cascales study could improve school climate in the intervention group and reduce the bullying of students ^34^.

Social support (i.e., informational, emotional, and instrumental, and appraisal support) *influence individuals social* function ^35^. Social support has an inverse relationship with involvement in bullying behaviors in students ^36, 37^. Our intervention to made change in students’ social support including networking and providing social support (informational, emotional and, instrumental) increased the social support score in the intervention group compared to the control group. Li et l increased social support of students through group discussion, role-playing, communication analysis, and sharing emotions^38^. Family, school and peers have a very important role in the development of social norms^39^. In Tunisia, from the perspective of most students, classmates and teachers take no action against bullying ^40^. In our study, the mean score of perceived social norms reduced significantly after intervention in the intervention group compared to the control group. In order to enhance this construct, we used group discussion about correct and incorrect beliefs related to bullying in pressure groups including parents, teachers and school staff. We tried to show these beliefs by role-playing of students and the consequences of their effects. The perceived social norms were improved in the students through posters with positive normed messages about bullying ^41^.

The results of this study should be interpreted with caution. First, although bullying is higher in deprived areas and in boys, focusing on deprived and male student population limits the generalizability of the results. Moreover, the self-reporting nature of the data collection may underestimate or overestimate the true effectiveness of the intervention. Therefore, we suggest further studies be conducted in areas with different socioeconomic characteristics and among female students. The results of our study using practical methods to reduce bullying can be useful for national and local policymakers in the education department and counseling sectors.

## Conclusion

The mean scores of SCT constructs have significantly improved after educational intervention in the intervention group. The amount of bullying and victimization reduced in the intervention group and social competence showed a significant increase in this group. Interventions focused on SCT can be effective in breaking the bullying cycle and improving social competence.

## Acknowledgements

This study is a part of PhD thesis and registered in IRCT (IRCT Cod: 20151112025014N3). We would like to appreciate all education authorities of Kermanshah City, teachers, school staff, parents and students who participated in this research.

## Conflict of interest

All authors have no conflict of interest to declare.

## Funding

The Vice-Chancellor of Research and Technology of the Hamadan University of Medical Sciences supported this study (Number: 9611107173, 30/01/2018).

## Highlights

- The anti-bullying intervention developed based on SCT and local sociocultural characteristics was effective in reducing students’ bullying and victimization behavior.
- The intervention improved the students’ social competence.
- Addressing key factors affecting the significant stakeholders strengthened the effectiveness of the intervention.
